# Acute Liver Failure and Dengue: Alcohol Matters

**DOI:** 10.4269/ajtmh.16-0985a

**Published:** 2017-03-08

**Authors:** Jose D. Debes, Ashraf Ashhab

**Affiliations:** 1 Department of Medicine, Infectious Diseases and International Medicine, University of Minnesota Minneapolis, MN and Department of Gastroenterology and Hepatology, Hennepin County Medical Center, Minneapolis, MN. E-mail: debes003@umn.edu; 2 Department of Medicine, Hennepin County Medical Center. Minneapolis, MN. E-mail: ashraf.a.ashhab@gmail.com

Dear Sir:

Acute liver failure secondary to dengue virus infection is a rare but catastrophic event, which is poorly understood. In the December issue of the Journal, Kye Mon and others describe liver-related complications among 1,926 patients with serologically confirmed dengue infection in Thailand.^1^ Although the study found only six individuals with acute liver failure, it serves to emphasize how lethal this complication can be. We are, nonetheless, surprised at the lack of assessment of alcohol intake in this study. The authors clarify in the article that none of the patients had a history of liver disease, but it is unclear whether alcohol intake was evaluated or how prior liver disease was assessed.

We explored other studies that reported acute liver failure with dengue infection, as summarized by Kye Mon and others; only one of these studies mentioned assessment of alcohol intake. This study indicated that “Although we attempted to gather information on prior alcohol consumption, the response rate was poor and the data collected were believed to be unreliable, precluding analysis.”^2^

Evaluating the presence of alcohol intake, either regularly or in binge, is critical whenever a provider addresses liver-related disease. In the case of acute hepatitis or liver failure from dengue infection, this seems particularly important. First, as the authors mention, hepatocytes and Kupffer cells are important targets for the virus.^3^ Acute and chronic alcohol intake induces mitochondrial toxicity in hepatocytes, affecting their metabolizing capacity. Moreover, recent studies suggest that binge drinking of alcohol induces bacterial translocation from the gut. This phenomenon usually triggers activation of Kupffer cells.^4^ The presence of bacterial translocation combined with dengue virus infection could induce a hyperactivation of Kupffer cells, with a significant worsening of the disease. Second, individuals with chronic alcohol intake could have unrecognized chronic liver disease, which can acutely worsen upon development of a viral infection, leading to acute on chronic liver failure, which shares a poor prognosis with acute liver failure, but requires a differential therapeutic approach.^5^ Third, alcohol consumption and abuse, once assumed to be a “western” habit, is now appreciated to be common in tropical countries, and likely has a deleterious effect on liver function and innate immune response in this setting.

We applaud the efforts of Kye Mon and others to shed light on a devastating aspect of dengue virus infection. Nonetheless, we believe that studies should better evaluate alcohol intake when addressing infection-related liver failure or hepatitis in tropical settings. This approach will likely clarify several aspects of this deleterious complication of viral infection and might emphasize a potential point of prevention for liver disease.
